# Effects of low-level laser therapy on stem cells from human exfoliated deciduous teeth

**DOI:** 10.1590/1678-775720150275

**Published:** 2016

**Authors:** Ana Paula FERNANDES, Marina de Azevedo JUNQUEIRA, Nádia Carolina Teixeira MARQUES, Maria Aparecida Andrade Moreira MACHADO, Carlos Ferreira SANTOS, Thais Marchini OLIVEIRA, Vivien Thiemy SAKAI

**Affiliations:** 1 - Universidade de São Paulo, Faculdade de Odontologia de Bauru, Departamento de Odontopediatria, Ortodontia e Saúde Coletiva, Bauru, SP, Brasil.; 2 - Universidade Federal de Alfenas, Departamento de Clínica e Cirurgia, Alfenas, MG, Brasil.; 3 - Universidade de São Paulo, Faculdade de Odontologia de Bauru, Departamento de Ciências Biológicas, Bauru, SP, Brasil.

**Keywords:** Lasers, Stem cells, Deciduous tooth

## Abstract

Low-Level Laser Therapy stimulates the proliferation of a variety of types of cells. However, very little is known about its effect on stem cells from human exfoliated deciduous teeth (SHED).

**Objective:**

This study aimed to evaluate the influence of different laser therapy energy densities on SHED viability and proliferation.

**Material and Methods:**

SHED were irradiated according to the groups: I (1.2 J/cm^2^ - 0.5 mW – 10 s), II (2.5 J/cm^2^ – 10 mW – 10 s), III (3.7 J/cm^2^ – 15 mW – 10 s), IV (5.0 J/cm^2^ – 20 mW – 10 s), V (6.2 J/cm^2^ – 25 mW – 10 s), and VI (not irradiated – control group). Cell viability was assessed 6 and 24 h after irradiation measuring the mitochondrial activity and using the Crystal Violet assay. Cell proliferation was assessed after 24, 48, and 72 h of irradiation by SRB assay.

**Results:**

MTT assay demonstrated differences from 6 to 24 hours after irradiation. After 24 h, groups I and IV showed higher absorbance values than those of control group. Crystal Violet assay showed statistically differences in the absorbance rate from 6 to 24 h after irradiation for groups III and VI. At 24 h after irradiation, Group III absorbance rate was greater than that of groups I, II, and IV. Group VI absorbance rate was greater than that of groups I and IV. SRB assay showed that the group I had higher rates than those of groups II, III, V, and VI, at 24 h after irradiation. After 48 h, group I exhibited the greatest cell proliferation rate followed by groups III, V, and VI. After 72 h, group III exhibited the lowest cell proliferation rate than those of groups II, IV, and V.

**Conclusions:**

The Low-Level Laser Therapy energy densities used in this study did not cause loss of cell viability and stimulated SHED proliferation within the parameters described in this study.

## INTRODUCTION

Tissue engineering with the triad of dental pulp stem cells, morphogens, and scaffolds may provide a useful alternative method for pulp-capping and root canal treatment^23^. The use of stem cells from human exfoliated deciduous teeth (SHED) might bring advantages for tissue engineering mainly because SHED was reported to have higher proliferation rate and increased cell population doublings, and they are retrieved from a tissue that is readily accessible in young patients. Additionally, previous studies proposed that dental pulp tissue engineering with stem cells could be ideally suited for dentin regeneration in response to noxious stimuli, such as caries^16^, and for young patients who have suffered pulp necrosis in immature permanent incisors as a consequence of trauma^8^.

The ability of Low-Level Laser Therapy (LLLT) to stimulate the proliferation of a variety of cell types has been considered as its most important physiological effect^27^. Studies have shown that LLLT promotes an increase in the proliferation rate of cells such as fibroblast^15,21^, endothelial cells^21^, osteoblasts^13^, epithelial cells^11^, and lymphocytes^6^. Concerning the proliferation of mesenchymal stem cells, a positive effect of LLLT on bone marrow^30^ and adipose tissue stem cells has been reported in literature^22^. However, very little is known about the effect of laser therapy on dental pulp stem cells. Therefore, this study aimed to evaluate the effect of LLLT on the viability and proliferation of SHED.

## MATERIAL AND METHODS

### Cell culture

Stem cells from human exfoliated deciduous teeth, gently provided by Dr. Bruno N. Cavalcanti (DDS, MSc, PhD, Institute of Science and Technology, São Paulo State University, São José dos Campos, SP, Brazil), were isolated by standard enzymatic digestion protocol and characterized according to Miura, et al.^20^ (2003) after Institutional Review Board approval (CAAE 02210312.1.0000.0077). The SHED were maintained in alpha modification of Eagle medium (MEMα, Invitrogen, Carlsbad, California) culture medium supplemented with 10% fetal bovine serum (FBS - Fetal Bovine Serum, Certified, Heat-inactivated, Gibco, Invitrogen, Grand Island, New York, United States) and 1% penicillin and streptomycin solution (Penicillin-Streptomycin, Gibco, Invitrogen, Grand Island, New York, United States). Cells were maintained in an incubator at 37°C and 5% CO_2_ and split at a ratio of 1:3 when they reached 80% of confluence. The medium was changed every two days. For all experiments, SHED at passages 4 to 8 was used.

### Experimental groups

The cultures were divided into six groups according to the LLLT energy density of irradiation: I (1.2 J/cm^2^- 0.5 mW – 10 s), II (2.5 J/cm^2^– 10 mW – 10 s), III (3.7 J/cm^2^– 15 mW – 10 s), IV (5.0 J/cm^2^– 20 mW – 10 s), V (6.2 J/cm^2^– 25 mW – 10 s), and VI (not irradiated – control group).

### Low-level laser therapy irradiation

Stem cells from human exfoliated deciduous teeth were seeded in 96-well plates (1x10^4^ cells/well) with alpha-MEM supplemented with 10% FBS and allowed to attach overnight. Prior to laser irradiation, the media of all wells were replaced by fresh culture medium supplemented with 1% FBS^9^, and then the plates were wrapped in a mask composed of black cardboard with holes located in the position of the wells of the experimental groups. Each hole was individually sealed by a hatch, also in black cardboard that allowed only the bore of the well that was being irradiated to remain open, while all the others were kept in the dark. The holes in the mask had the diameter of 0.5 cm^2^, while the laser spot had the diameter of 0.4 cm^2^. LLLT irradiation was made through the transparent bottom of the 96-well plates, keeping the distance between the light beam and the cell monolayer constant at 1 mm. Therefore, the radiation passed directly to the cell monolayer via the plate base without travelling through the culture medium reaching the cells, following the methodology previously adopted^3,11,29^. The power of the laser was measured with the Lasercheck PowerMeter (Coherent Inc., Santa Clara, California) prior to each application. Aluminium-gallium-indium-phosphide (InGaAlP) Low-level Lase ility (MTT assay) of SHED regardi (Twin Flex Evolution MMOptics^®^ – São Carlos, SP, Brazil) was employed in all groups at wavelength of 660 nm (red); output beam area of 0.04 cm^2^, and varying the energy density in function of the power used for each experimental group. The control group was treated under identical conditions except that the laser device was kept off. After irradiation, the media of all wells were replaced by fresh culture medium supplemented with 10% FBS.

### MTT assay

Aiming to evaluate the ility (MTT assay) of SHED regardicytotoxicity of the different LLLT irradiation densities, 3-(4, 5-dimethyl-2-thiazolyl) -2, 5-diphenyl -2H- tetrazolium bromide (MTT) assay was performed after 6 and 24 hours of irradiation. At the end of the respective incubation time, the supernatants were discarded and 200 µL MTT solution was added into each well to a final concentration of 5 mg/mL. After an additional 4-hour incubation period, the supernatant was discarded, and 200 µL dimethyl sulfoxide (DMSO, Fisher Scientific, Hampton, New Hampshire, United States) was added to solubilize the formazan crystals. Immediately, the absorbance was read in a microplate reader (Zenyth, 200 RT, Anthos) at 560 nm^7^. Data were obtained from three wells per condition.

### Crystal violet assay

The crystal violet assay is useful for obtaining quantitative information about the relative density of cells adhering to multiwell cluster plates^12^. Crystal violet solution is capable of assessing cellular viability because it stains cellular DNA of living cells. SHED were irradiated and then incubated at 37°C, 5% CO_2_, and divided into 1:3 rates when reaching to 80% confluence. The medium was changed every two days. At the end of incubation, supernatants were discarded and crystal violet solution was added into the wells. Then, the plates were incubated at 37%, 5% CO_2_ for 6 and 24 h. Cell platting was similar to that of the MTT assay regarding steps, periods, and cell amounts. All culture media was removed from the plate, including the Blank and control groups. Next, each well was washed with PBS (200 uL). Following, 100% methanol (200 uL) was used for 10 min. Elapsed that time, methanol was removed and crystal violet solution (200 uL) was added to the wells for 3 min. Following, crystal violet solution was removed, and each well was washed with PBS 1x (200 uL) twice. Then, sodium citrate 0.05 mol.L (200 uL) was added for 10 min. The absorbance reading was performed in spectrophotometer at 570 nm wavelength. Negative controls (Blank) were wells without cells.

### SRB assay

Cell proliferation by Sulforhodamine B (SRB) assay was assessed after 24, 48, and 72 hours of irradiation. At the end of the respective incubation time, cells were fixed by the addition of 10% trichloroacetic acid and incubated for 1 hour at 4°C. Plates were washed in tap water five times and allowed to dry. Cellular protein was stained by adding 4% SRB in 1% acetic acid and incubated at room temperature for 30 minutes. Excess SRB was removed by washing the wells with 1% acetic acid and remaining SRB was solubilized in 10 mM Tris-base unbuffered. Absorbance was determined on a spectrophotometer at a wavelength of 565 nm^18,26^. Data were obtained from three wells per condition.

### Statistical analysis

Statistical analysis was performed using R Statistical Software. Viability and proliferation data were analyzed by two-way analysis of variance (ANOVA), followed by Tukey’s test. Differences were considered significant at p<0.05. Data were expressed as mean value and standard deviation.

## RESULTS

### MTT assay

**Figure 1:**
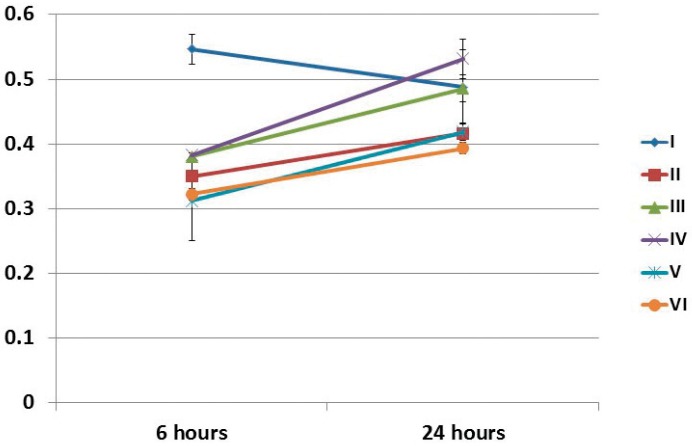
Cell viability (MTT assay) of SHED regarding the time after irradiation and experimental groups. I (1.2 J/cm2 - 0.5 mW – 10 s), II (2.5 J/cm2 – 10 mW – 10 s), III (3.7 J/cm2 – 15 mW – 10 s), IV (5.0 J/cm2 – 20 mW – 10 s), V (6.2 J/cm2 – 25 mW – 10 s), and VI (not irradiated – control group)

All groups except for group I showed statistically significant differences in the absorbance rate from 6 to 24 hours after irradiation. At 6 hours after irradiation, group I exhibited the greatest absorbance value as compared with those of the other groups. At 24 hours after irradiation, both group I and IV showed higher absorbance values than that of the control group (VI). Group IV also showed higher absorbance value than those of groups II and V, and this difference was statistically significant (p<0.05) (Figure 1).

### Crystal violet assay

**Figure 2:**
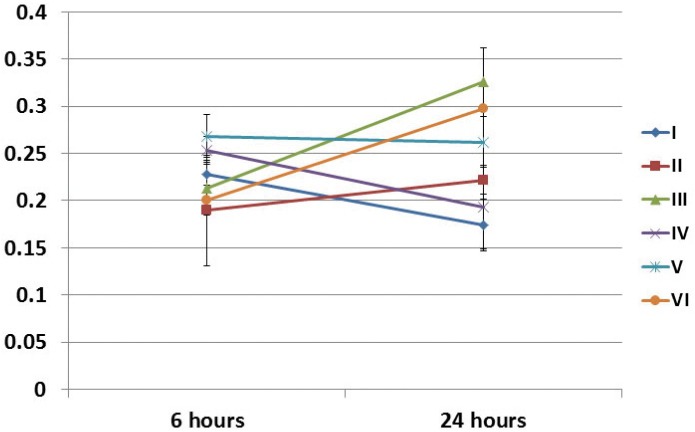
Cell viability (Crystal violet assay) of SHED regarding the time after irradiation and experimental groups. I (1.2 J/cm2 - 0.5 mW – 10 s), II (2.5 J/cm2 – 10 mW – 10 s), III (3.7 J/cm2 – 15 mW – 10 s), IV (5.0 J/cm2 – 20 mW – 10 s), V (6.2 J/cm2 – 25 mW – 10 s), and VI (not irradiated – control group)

Groups III and VI showed statistically significant differences in the absorbance rate from 6 to 24 hours after irradiation. At 6 hours after irradiation, there was no statistical difference between groups. At 24 hours after irradiation, group III absorbance rate was greater than that of groups I, II, and IV. The positive control absorbance rates were greater than those of groups I and IV (Figure 2).

### SBR assay

**Figure 3:**
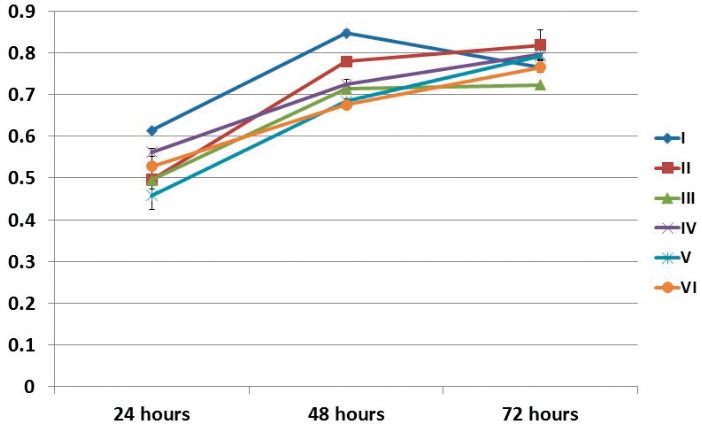
Cell proliferation (SRB assay) of SHED regarding the time after irradiation and experimental groups. I (1.2 J/cm2 - 0.5 mW – 10 s), II (2.5 J/cm2 – 10 mW – 10 s), III (3.7 J/cm2 – 15 mW – 10 s), IV (5.0 J/cm2 – 20 mW – 10 s), V (6.2 J/cm2 – 25 mW – 10 s), and VI (not irradiated – control group)

At 24 hours after irradiation, group I showed statistically significant higher cell proliferation than groups II, III, V, and VI. At 48 hours after irradiation, group I exhibited the greatest cell proliferation compared with the other groups, and group II showed higher cell proliferation than groups III, V, and VI, with statistically significant difference. At 72 hours after irradiation, group III exhibited statistically significant lower cell proliferation than groups II, IV, and V (Figure 3).

## DISCUSSION

The biostimulatory effect of LLLT has been reported for at least four decades. Since this effect was first reported, researchers in this field have sought to determine the best laser protocol to promote the reported positive biostimulatory effects attributed to low energy irradiation. However, studies investigating this effect of LLLT on dental stem cells are scarce in the literature and there are no reports on stem cells from human exfoliated deciduous teeth. By submitting cells to LLLT, some authors reported higher proliferation rates of gingival fibroblasts^9^, dental pulp stem cells from permanent teeth^10^, and stem cells derived from bone marrow and adipose tissue^4,27^. It has been suggested that the energy of the laser is absorbed by intracellular chromophores and converted into metabolic energy, which is then used by the mitochondrial respiratory chain to produce ATP and increasing DNA activity and the synthesis of RNA and proteins.

AlGhamdi, et al.^1^ (2012) affirmed that, for biological effects, laser wavelengths may be red or near infrared (600–1200 nm), and for biostimulatory effects, energy fluences may range from 0.05 to 10 J/cm^2^ to induce cell proliferation, whereas energies greater than this value (>10 J/cm^2^) may promote antiproliferative effects. In this present study, the wavelengths were set at 660 nm (red laser) and different energy densities were established in relation to the Irradiation Time in function of the Power, according to the experimental group. The energy density may be important to reach an improvement in cell growth^25^. Notwithstanding, the effect of energy density variation is still unknown, mainly on pulp cells of human primary teeth. Many studies have tested different energy densities^1,5,14^ and, according to some authors, the energy densities ranging from 0.5–4 J/cm^2^ have been more effective in stimulating cellular growth^1,2,5,19,24,29^. According to Karu, et al.^17^ (1987), the dose increase damages photoreceptors, which reduces the biomodulatory effect of the laser as a result of the inhibition of metabolism and consequent cell death. In this study, for MTT assay, the lowest dose, group I (1.2 J/cm^2^), showed the more favorable results on cell viability as much as 6 to 24 hours after irradiation compared with the others groups. For Crystal Violet assay, the group III (3.7 J/cm^2^) showed the more favorable results at 24 hours after irradiation.

Concerning cell proliferation, group I also exhibited the best cell proliferation rate mainly at 24 and 48 hours after irradiation compared with that of the control group. The group V was the group with the result more similar to that of the non-irradiated group. The results are in agreement with the studies of Barboza, et al.^4^ (2014), who irradiated bone marrow and adipose tissue derived from mesenchymal stem cells, and Stein, et al.^28^ (2005), who irradiated human osteoblasts. Both authors had higher cell proliferation using a wavelength of 670 nm and a dose of 1.0 J/cm^2^ at the first 72 hours after irradiation.

Regarding the concern of the appropriate serum, additional Crystal Violet assay was performed in order to compare cell viability after irradiation of SHED growing in medium supplemented with either 1% FBS or 10% FBS. After 6 hours of irradiation, the absorbance rates of cells growing in the different serum concentration media were statistically similar for all the irradiation doses evaluated. However, after 24 hours, higher values were observed for those cells growing in medium supplemented with 1% FBS in comparison with 10% FBS for all of the doses (data not shown). These data support our experiments with the low concentration of FBS, which seems to be the most appropriate serum concentration to show the effect of laser phototherapy in SHED. The mechanism of cellular stress occurs because of alterations triggered by oxidant agents, temperature, and nutritional deficiency of the culture medium. The decrease in the concentration of the fetal bovine serum in the culture medium (nutritional deficit) has been efficiently used to evaluate the effects of low level laser irradiation on cellular metabolism^2,9,10^. In this present study, after plating on Alpha Modification of Eagle medium supplemented with 10% FBS, the cells were incubated for 24 h until reaching a subconfluent state in the wells. Elapsed that period, all wells received a medium supplemented with 1% FBS, in such a way that the cells reach the quiescence state stage at the moment of the irradiation^9^.

The study of Ginani, et al.^14^ (2015) evaluated the literature on the effect of low level laser on stimulating mesenchymal stem cells from 2002 to 2013 and found that the laser parameters ranged in relation to the wavelength both in visible (red) and invisible (infrared) light, but no study compared the effects of the two wavelengths (red and infrared) on cellular proliferation. Concerning the dose, most of the studies used 0.5 J/cm^2^, ranging from 0.05 J/cm^2^ to 42 J/cm^2^. The laser power varied in the different light spectrum: for visible light, power ranged from 0.02 mW to 119 mW; for invisible light from 50 mW to 800 mW. The effect of low level laser intensity has been evaluated on the proliferation of the mesenchymal stem cells of different tissues: bone marrow, dental pulp, and periodontal ligament. The authors concluded that greater proliferation occurred in mesenchymal stem cells from bone marrow and adipose tissue after applying low level laser, but further studies on mesenchymal stem cells from dental tissues are necessary. Finally, it is necessary to standardize the low level laser parameters, improving not only the comparison of the results but also the results themselves. Basso, et al.^5^(2012) affirmed that determining the parameter and ideal techniques of irradiation is mandatory to develop further studies to verify the potential and adequate biostimulatory effect of LLLT. Consequently, the knowledge of the proper combination of these parameters (e.g., wavelength, power density, and energy density) is required to reach the desirable effects of the treatments.

The search for solutions and ideal materials in any knowledge area in dentistry should be targeted to know and indicate biocompatible therapies, aiming at repair processes and providing the natural and biological pulp regeneration. The recent advancement in the cellular and molecular field provided a better understanding of the alterations and behavior of the pulp tissue during the tissue repair process, enabling to biologically evaluate the different strategies for pulp therapy. Notwithstanding, few evidences demonstrate that LLLT would have a directly influence on SHED.

In summary, the present results showed that LLLT using different energy densities has a positive influence on the *in vitro* viability and proliferation rates of SHED and may be a useful tool for tissue engineering using stem cells. However, further studies are needed to standardize the laser parameters to improve the yield of cells in culture as SHED. The biostimulation of dental pulp stem cells might be an important tool for regenerative therapy and tissue engineering^1,14^.

## CONCLUSION

The Low-Level Laser Therapy energy densities used in this study did not cause loss of cell viability and stimulated SHED proliferation within the parameters described in this study.
